# Equity of access to maternal health interventions in Brazil and Colombia: a retrospective study

**DOI:** 10.1186/s12939-018-0752-x

**Published:** 2018-04-11

**Authors:** Amaila De La Torre, Zlatko Nikoloski, Elias Mossialos

**Affiliations:** Department of Health Policy, London School of Economic and Political Science, Houghton Street, London, WC2A 2AE UK

**Keywords:** Maternal mortality, Inequity, Access to healthcare, Concentration index, Brazil, Colombia

## Abstract

**Background:**

Reducing maternal mortality is a top priority in Latin American countries. Despite the progress in maternal mortality reduction, Brazil and Colombia still lag behind countries at similar levels of development.

**Methods:**

Using data from the Demographic Health Survey, this study quantified and compared, by means of concentration indices, the socioeconomic-related inequity in access to four key maternal health interventions in Brazil and Colombia. Decomposition analysis of the concentration index was used for two indicators – skilled attendance at birth and postnatal care in Brazil.

**Results:**

Coverage levels of the four key maternal health interventions were similar in the two countries. More specifically, we found that coverage of some of the interventions (e.g. ante-natal care and skilled birth assistance) was higher than 90% in both countries. Nevertheless, the concentration index analysis pointed to significant pro-rich inequities in access in all four key interventions in both countries. Interestingly, the analysis showed that Colombia fared slightly better than Brazil in terms of equity in access of the interventions studied. Finally, the decomposition analysis for the presence of a skilled attendant at birth and postnatal care in Brazil underlined the significance of regional disparities, wealth inequalities, inequalities in access to private hospitals, and inequalities in access to private health insurance.

**Conclusions:**

There are persistent pro-rich inequities in access to four maternal health interventions in both Brazil and Colombia. The decomposition analysis conducted on Brazilian data suggests the existence of disparities in system capacity and quality of care between the private and the public health services, resulting in inequities of access to maternal health services.

**Electronic supplementary material:**

The online version of this article (10.1186/s12939-018-0752-x) contains supplementary material, which is available to authorized users.

## Background

Maternal deaths received a high level of global political attention for the first time in 2000, when United Nations member states pledged a reduction of 75% in the 1990 Maternal Mortality Ratio (MMR) by 2015 as part of their commitment to achieve eight Millennium Development Goals (MDG). Moreover, the new commitments set by the Sustainable Development Goals (SDG) in 2015 aim to build on the efforts that led to significant improvements in maternal health indicators. More specifically, SDG 3.1 sets a goal to decrease the global MMR to less than 70 per 100.000 live births by 2030.

Despite these commitments, women continue to die owing to pregnancy-related causes —particularly in low- to middle-income countries, and mostly during labour, delivery, or within 24 h postpartum [1]. A large body of research suggests that MMR is mostly due to direct obstetric causes (e.g. haemorrhage, obstructed labour, infections, and hypertensive diseases) [2, 3]. However, most causes of MMR could be reduced with timely access to quality health care [2, 4]. As most of the maternal deaths occur in the poorest countries in the world (and among the poorer socioeconomic segments of the developed world), a higher MMR violates the principle of horizontal equity [5]. Strategies aimed at reducing maternal deaths need to address inequities in access to good quality maternal health interventions. Existing evidence singles out four key interventions relevant for maternal health: skilled birth assistance (SBA), Caesarean section (C-section), postnatal care (PNC), and antenatal care (ANC). The interventions SBA, C-section, and PNC are associated with the timing of most maternal deaths, which are “clustered around labour, delivery and the immediate postpartum period” [6]. ANC is considered essential in making behavioural changes that have a positive influence on maternal health outcomes (e.g. promoting prevention and self-care behaviours) [7, 8].

### Equity of access to maternal health interventions in Brazil and Colombia

Maternal mortality has also received significant attention in Latin American and Caribbean regions, which encompass countries at various levels of development and with different health system structures. Brazil and Colombia are two of the biggest countries in the region that follow divergent healthcare financing and provision models. Structural reforms in Brazil in the late 1980s, enshrined in the Brazilian constitution, aimed at achieving universal healthcare coverage. Nevertheless, chronic underfunding coupled with management weakness, has constrained improvements in the quality of services and access to healthcare services [9]. A set of structural reforms in the Colombian healthcare system initiated in the early 1990s had similar aims, though existing evidence indicates that universal healthcare coverage was not achieved until 2011 [10]. Despite these achievements, a significant part of both the Brazilian and Colombian population has supplementary private health insurance. A report by the World Health Organization (WHO) suggests that supplementary private health insurance represents 20.9% and 11.9% of total healthcare expenditures in Brazil and Colombia, respectively [11]. Bearing in mind these two different contexts, both Brazil and Colombia implemented several policies with a specific objective of increasing coverage and enhancing the quality of maternal health services. The aim of these policies was to improve maternal and neonatal health outcomes (Additional file 1: Table S1).

Maternal health has been a component of the Brazilian health agenda before the initiation of reforms aimed at achieving universal health coverage. However, it was not until the watershed moment of the ‘National Programme for the Humanization of Antenatal - Delivery and Post-Partum Care’ (2000) that maternal health became a political priority. The aim of the programme was to achieve significant access, coverage and quality of key maternal health interventions by anchoring maternal health services to the principles of sexual and reproductive rights and, in turn, position such services as a human right. Despite these ambitious goals, the programme has had limited success [12]. Several other policy initiatives such as the ‘Pact for the Reduction of Maternal and Newborn Mortality’ (2004) and the ‘Stork Network Strategy’ (2011) are evidence of the unfinished agenda in maternal health in Brazil. However, all those programmes and initiatives are struggling with the issues of quality in antenatal, childbirth, and postnatal care; system capacity limitations; and lack of integration among maternal health services [13–15].

In Colombia, several directives issued by the Ministry of Health and Social Protection after the 1993 Health System Reform sought to raise the standard of care for maternal health interventions. However, it was only in 2003 that a comprehensive policy for maternal health was enacted. The 2003 Sexual and Reproductive Health Policy approached maternal health issues in the broader context of women’s lifecycle, recognizing that sexual and reproductive rights were human rights that needed to be upheld in order to protect the life of women, mothers and children. This represented an important change in the paradigm; however, implementation difficulties led the government to propose an emergency plan, ‘Crash Plan’ in 2004; this plan was expected to expedite progress in the reduction of maternal deaths. Despite significant efforts to improve policies focused on maternal health outcomes, those responsible for diagnosis, policy analysis, and policy design often came to the same conclusion: that the stagnation in reductions of maternal deaths, despite high levels of coverage achieved with key maternal health interventions, pointed to issues of quality of care [16, 17].

Despite recent efforts, MMRs in Brazil and Colombia are considered high and lag behind Latin American countries at a similar level of economic development (Mexico and Argentina) [18]. Moreover, the improvements in maternal health fell short of the MDG target 5 (improve maternal health), therefore MMR of the two countries remains unacceptably high [18]. Against this background, our study had a three-fold objective: (i) to analyse the equity of access to four key maternal healthcare interventions: (a) ANC of at least four visits; (b) SBA, which for both Brazil and Colombia encompasses doctors and nurses given there is no professional midwifery; (c) C-section; and (d) PNC; (ii) to compare the equity of access Brazil and Colombia; and (iii) to further study some of the drivers of inequitable access, while paying particular attention to private health insurance in Brazil.

## Methods

### Data sources and definition of healthcare intervention variables

We relied on the last available Demographic Health Surveys (DHS) (Brazil [2006] [19] and Colombia [2010]) [20]. The definitions of the coverage indicators for the four key maternal healthcare interventions are presented in Table 1 and are based on the paper by Ronsmans et al. (2008) [6].

**Table 1:** Coverage Indicator definitions

Indicator name	Indicator description	Numerator	Denominator
ANC: Antenatal care (4 or more visits)	Percent of women (counted for each pregnancy) attended at least four times during pregnancy by any provider (skilled or unskilled) for reasons related to the pregnancy in the 5 years prior to the survey	Number of women (counted for each pregnancy) attended at least four times during pregnancy by any provider (skilled or unskilled) for reasons related to the pregnancy in the 5 years prior to the survey	Total number of women (counted for each pregnancy) between 15 and 49 years who had a live birth in the 5 years prior to the survey
SBA: Skilled birth assistance	Percentage of live births attended by skilled health personnel (only doctor and nurse are considered skilled attendants as there is no proffesional midwifery in Brazil and Colombia)	Number of live births in the 5 years prior to the survey attended during delivery by skilled attendants (doctor or nurse)	Total number of live births to women aged 15–49 years in the 5 years prior to the survey
C-section rate	Percentage of live births delivered by Caesarean section	Number of live births in the 5 years prior to the survey delivered by Caesarean section	Total number of live births to women aged 15–49 years in the 5 years prior to the survey
PNC: Postnatal care for mothers	Percentage of women (counted for each pregnancy) who had a postnatal care consultation within two months of childbirth	Number of women (counted for each pregnancy) who had a postnatal care consultation within two months of childbirth (regardless of place of delivery) in the 5 years prior to the survey	Total number of women aged 15–49 years (counted for each pregnancy) who had a live birth in the 5 years prior to the survey (regardless of place of delivery)

Source: TRACKING PROGRESS IN MATERNAL, NEWBORN & CHILD SURVIVAL THE 2008 REPORT. Changes were made to numerators and denominators of the ANC and PNC to account not only for the women but to each of the woman pregnancies in the 5 years prior to the survey

### Measures of inequality (concentration index) and decomposition analysis

In analysing equity in access, we relied on the standard concentration index (CI) methodology. We selected this measure as it lends itself to decomposition into the determinants of inequality. The CI is a summary measure of the degree of inequality of distribution of the variable of interest that places equal weights on the different degrees of inequalities along the income distribution [21]. It can be expressed as follows [22]:

1 $$ \mathrm{C}=\frac{2}{\mu }{\sum}_{t=1}^T{f}_t{\mu}_t{R}_t-1, $$

where C is the CI, $\mu ={\sum}_{t=1}^T{f}_t{\mu}_t$ expresses the overall mean quantity of the health related “good” (i.e. health intervention), *μ*_*t*_ is the mean coverage rate of the *t*_*th*_ socioeconomic group, and *R*_*t*_ is the relative rank of the socioeconomic group along the socioeconomic distribution of the total population. The CI is a summary measure of the degree of inequality of distribution of the health-related “good”, and it is bounded between − 1 and + 1, where 0 is its minimum value (reflecting equality) and − 1 and + 1 are its maximum possible values, where − 1 corresponds to a distribution that favours the poor and + 1 corresponds to a distribution that favours the rich [21].

The CI approach to measure inequalities in health sector variables has several advantages [21, 23, 24]. First, it provides a measure of the variations of inequality across the entire income distribution. Second, the CI is a summary measure that provides a numerical measure of inequality; when this is required, it facilitates inter-temporal and cross-country comparisons of levels of socioeconomic-related inequality. Third, the CI allows the calculation of standard errors to check statistical significance of results derived from survey data with different sample sizes and designs. Finally, the CI lends itself easily to decomposition into the determinants of inequality.

Despite these advantages, the CI approach is associated with certain shortcomings, such as the ‘bounds issue’ for bivariate variables. The CI inter-temporal and cross-country comparability is limited when the health care variable of interest is a bivariate variable. The existing literature argues that concentration indices for bivariate variables are bounded by the means of the variables; that is, equal concentration indices of two countries that have different mean rates of utilization of a given service reflect different levels of inequity in access, because “the mean of the distribution places bounds on the possible values of the concentration index” [25]. Thus, minimum and maximum values of the CI are no longer (− 1, + 1), but (μ − 1 − μ) respectively. One way to approach this issue is to normalize the CI dividing it by 1 − μ [21]. We followed this approach in this study.

Women were ranked according to socioeconomic status of the household in which they lived, and the sample was then divided into quintiles. For each quintile, coverage indicators were calculated. The chosen measure to capture the socioeconomic status of households was a wealth index, based on the principal-components analysis [26]. While the Colombian DHS contains a wealth index already calculated by the survey providers, to make the results comparable between the two countries, we constructed a similar index (based on the same components) for Brazil (details of this exercise, including limitations, are reported in Additional files 2 and 3).

The CI analysis was coupled with a standard decomposition analysis of the socioeconomic-related inequality affecting access. Socioeconomic-related inequality affecting a health variable of interest (captured by the CI) can be expressed as the result of the socioeconomic related inequalities of its determinants. Given the problems associated with the usage of linear models in conducting the decomposition analysis, we relied on a methodology for the decomposition analysis that used a probit model and its ‘partial effects’ [27, 28].

The general model is given by Eq. (2) below:

2 $$ E\left({y}_i|{x}_i\right)=G\left({\sum}_k{\beta}_k{x}_i^k\right) $$

where G represents the functional form for a non-linear model. As proposed by van Doorslaer, we have ‘restore [d] the mechanics of the decomposition framework by replacing the *β*_*k*_ parameters in equation by the ${\beta}_k^m$ parameters”, where the ${\beta}_k^m$ represent the “partial effects’ of the x (the determinants of y) in the linear approximation of the non-linear model expressed by Eq. (3):

3 $$ {y}_i={\sum}_k{\beta}_k^m{x}_i^k+{u}_i $$

Consequently, we conducted a decomposition analysis of the socioeconomic-related inequality affecting access to SBA and access to PNC in Brazil using a probit model with its marginal effects. The concentration indices for these two interventions were calculated again for microdata [29]. We have selected these two measures for the decomposition analysis, as the existing research on the epidemiology of maternal mortality suggests that most maternal deaths occur during labour, delivery, or the first 24 h postpartum. Discovering the reasons behind the inequities in access to SBA and PNC could provide further evidence concerning why Brazil was unable to make progress on the reduction of maternal deaths.

For the decomposition analysis, the dependent variable (i.e. access to the two key maternal health interventions, SBA and PNC) was explained as a function of need (pregnancy status and demographic characteristics, such as age); certain predisposing factors (whether the pregnancy was planned or wanted, total number of live births, marital status, and race); and enabling resources (the mother’s educational attainment, the mother’s wealth index, having a complementary private health insurance plan, being—or not—a beneficiary of the cash-transfer program *‘Bolsa Familia’* (a conditional cash transfers programme targeted towards the poor and vulnerable, which, inter alia, serve as a proxy for social status), and—as a proxy for community level factors—the region and the location of the residence in a rural or urban area), following the behavioural model of health service use [30]. Deliveries in a private hospital any by a doctor were considered additional determinants of SBA and PNC, respectively.

Colombian DHS did not allow us to combine child and mother datasets, which could have been used to run linear regression models to decompose SBA and PNC. For this reason, we focused on Brazil. All analyses were conducted using STATA (version 13.0). For all calculations, the sample weights related to the survey design were taken into account.

## Results

### Coverage rates of maternal health interventions and the extent of inequality across interventions and countries

**Fig. 1:**
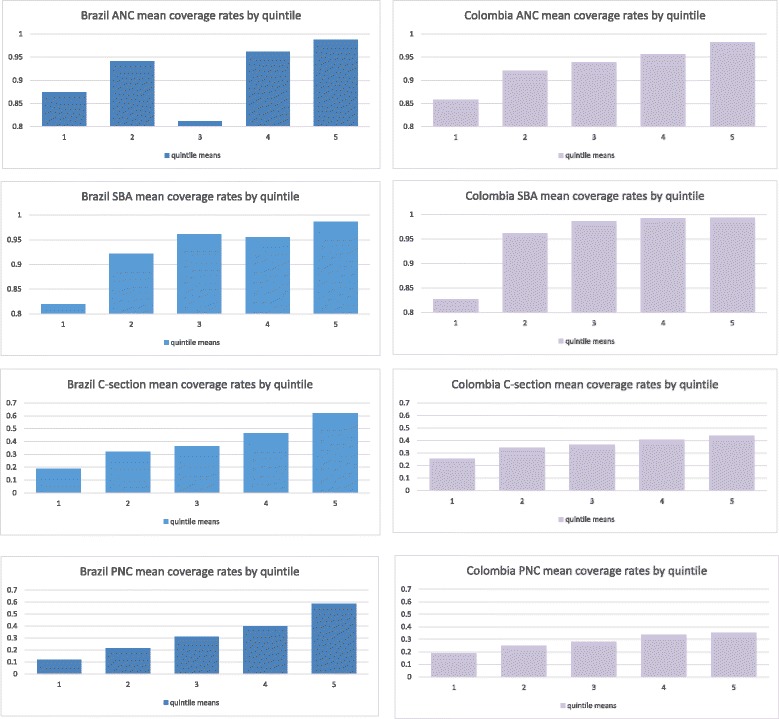
Intervention mean coverage rates by quintile Coverage rates in the vertical axes ranges from 0 to 1 however to facilitate visual presentation only part of the scale was shown (from 0 to 0,8 or from 0,8 to 1) to highlight differences in coverage across quintiles. SBA, skilled birth assistance; PNC, postnatal care

Table 2 provides a summary of coverage rates for each of the four key maternal health interventions in each country. Figure 1 provides a visual representation of mean coverage rates in each country by quintile for each intervention. ANC and SBA displayed average coverage levels above 90% in both countries. PNC coverage mean rates were below 50% in both countries, while the mean C-section rate for the two countries were well above the 15% threshold recommended by WHO [31]. Overall differences in mean coverage rates between Brazil and Colombia were not significant.

**Table 2:** Interventions coverage based on wealth quintile

Interventions	Brazil						Colombia					
	Quintiles						Quintiles					
	1	2	3	4	5	Overall mean coverage rate	1	2	3	4	5	Overall mean coverage rate
ANC (4 visits or more)	87.4%	94.1%	81.1%	96.1%	98.7%	91.3%	85.8%	92.1%	93.9%	95.7%	98.3%	92.8%
SBA	81.9%	92.2%	96.1%	95.5%	98.6%	92.9%	82.7%	96.1%	98.7%	99.3%	99.4%	94.3%
C-section	18.7%	32.2%	36.4%	46.2%	62.1%	39.1%	25.4%	34.1%	36.4%	40.6%	43.9%	35.0%
PNC (within 2 days of birth)^a^							1.1%	1.1%	1.2%	1.3%	1.8%	1.2%
PNC (within 2 months after birth)	11.6%	21.5%	31.2%	39.9%	58.6%	32.5%	18.7%	24.9%	27.9%	33.5%	35.3%	26.9%

*SBA* skilled birth assistance, *PNC* postnatal care

^a^Only the Colombian DHS (2010) asked for attendance of postnatal care within 2 days of delivery

### Concentration index

Table 3 provides a summary of the concentration indices, the normalized concentration indices, the standard errors, t-values, and 95% confidence intervals. Inequities in access to all interventions in the two countries favoured the rich.

**Table 3:** Concentration indices and key statistics

Interventions	Brazil							Colombia						
	C	Mean coverage rates (μ)	C normalized = (C/1 - μ)	SE (C)	*t* (C)	Low	High	C	Mean coverage rates (μ)	C normalized = (C/1 - μ)	SE (C)	*t* (C)	Low	High
ANC (4 visits or more)	0.022	0.91	0.506	0.013	1.70	-0.004	0.048	0.024	0.93	0.329	0.008	3.13	0.008	0.039
SBA	0.032	0.93	0.836	0.013	2.37	0.005	0.059	0.035	0.94	0.612	0.015	2.36	0.005	0.065
C-section	0.207	0.39	0.362	0.055	3.74	0.095	0.318	0.097	0.35	0.150	0.029	3.30	0.038	0.157
PNC (within 2 months after birth)	0.277	0.32	0.456	0.068	4.05	0.14	0.415	0.123	0.27	0.168	0.028	4.32	0.066	0.180

*SE* standard errors, t (C) = t-values and Low and High are the 95% confidence intervals limits

The t variable for C is defined as t = C/SE(C)

The critical value t_α/2_ of the variable t for the confidence intervals is obtained from the t distribution for α/2 (2, 5%) level of significance and 38–40 df (degrees of freedom)

The 95% confidence interval for C is given by: C ± t_α/2_ SE (C)

*SBA* skilled birth assistance, *PNC* postnatal care

When first considering non-normalized CI, we found that inequity levels in both countries were similar. Further, ANC and SBA were the most equitable interventions (both of which had a mean coverage rate above 90%). The most inequitable intervention in Brazil was PNC. Although PNC in Colombia was more equitable than that in Brazil, PNC was the most inequitable maternal health intervention in Colombia. Once normalization was considered, the overall findings changed the dimension of inequity, not the presence or absence of inequity. Greater changes were observed for those interventions with the highest mean levels of coverage in the two countries after normalization. Before normalization, CI results indicated almost no inequity for ANC and SBA, as CI was close to zero.

The most inequitable intervention was SBA in Brazil, followed by SBA in Colombia, ANC in Brazil, and PNC in Brazil. On the contrary, our study found that C-section mean coverage rates are well above the 15% threshold recommended by WHO in the two countries [31]. And even for the poorer groups of the population, both in Brazil and Colombia, the C-section coverage rate was higher than the 15% threshold. Hence, the uneven distribution of caesarean deliveries across the groups, ranked according to their socioeconomic status, does not seem to indicate that there are access issues to medically prescribed caesarean delivery. While at first these results suggest the existence of inequality, it should not be considered as such, given that C-sections are not recommended in most cases.

### Decomposition analysis of SBA and PNC in Brazil

**Fig. 2:**
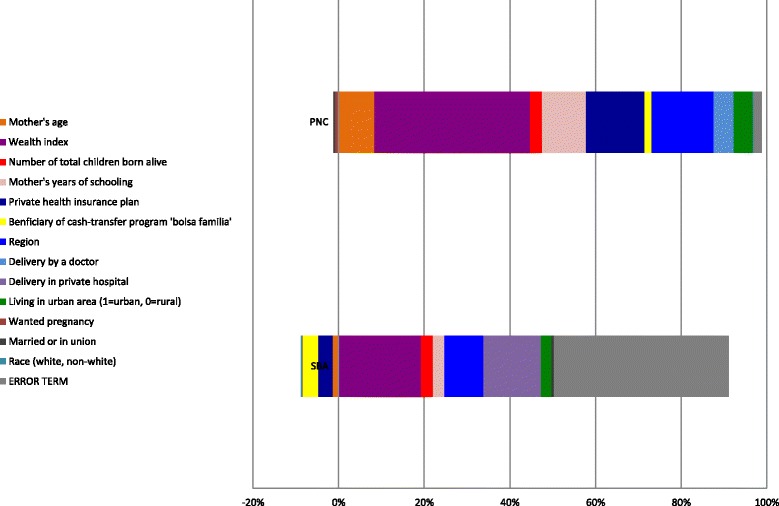
Decomposition analysis for PNC and SBA in Brazil

Table 4 provides an overview of the contributions to socioeconomic-related inequality in access to SBA and PNC (within 2 months of birth) for each of the variables considered as determinants of access in the probit regression models. Figure 2 provides a visual presentation of the percentage contributions to inequity of the main determinants of access to SBA and PNC.

**Table 4:** Inequality decompositions for SBA and PNC (within 2 months after birth)

	SBA					PNC				
	Elasticities	Concentration indices	Contributions to C	Contributions to C (%)	*P* values	Elasticities	Concentration indices	Contributions to C	Contributions to C (%)	*P* values
Mother’s age	−0.0082	0.0429	−0.0004	−1.70%	0.618	**0.5588**	**0.0422**	**0.0236**	**8.59%**	**0.006**
Wanted pregnancy	−0.0004	0.0702	0.0000	−0.13%	0.837	−0.034	0.068	−0.002	− 0.86%	0.295
Number of total children born alive	**0.0088**	**−0.0774**	**0.0007**	**3.33%**	**0.020**	−0.1018	−0.0769	0.0078	2.85%	0.114
Married or in union	0.0039	0.0396	0.0002	0.75%	0.397	−0.0293	0.0387	−0.0011	− 0.41%	0.711
Race (white, non-white)	−0.0004	0.1924	−0.0001	−0.39%	0.769	0.0036	0.1912	0.0007	0.25%	0.876
Mother’s years of schooling	0.0051	0.1333	0.0007	3.30%	0.356	**0.2170**	**0.1327**	**0.0288**	**10.49%**	**0.014**
Wealth index	**0.0193**	**0.2479**	**0.0048**	**23.44%**	**0.003**	**0.4136**	**0.2473**	**0.1023**	**37.29%**	**0.000**
Private health insurance plan	−0.0017	0.4892	−0.0008	−4.13%	0.341	**0.0797**	**0.4817**	**0.0384**	**14.00%**	**0.000**
Benficiary of cash-transfer program ‘bolsa familia’	**0.0026**	**−0.3469**	**−0.0009**	**−4.44%**	**0.004**	−0.0140	− 0.3432	0.0048	1.76%	0.467
Delivery in private hospital	**0.0060**	**0.5611**	**0.0034**	**16.40%**	**0.000**					
Delivery by a doctor						**0.2118**	**0.0623**	**0.0132**	**4.81%**	**0.035**
Region	**0.0096**	**0.2345**	**0.0023**	**11.06%**	**0.001**	**0.1730**	**0.2344**	**0.0405**	**14.78%**	**0.000**
Living in urban area (1 = urban, 0 = rural)	**0.0069**	**0.0875**	**0.0006**	**2.95%**	**0.043**	**0.1423**	**0.0883**	**0.0126**	**4.58%**	**0.013**
Concentration Index w/micro data			0.0103					0.2692		
		0.0204	50.4%				0.2743	98.12%		
Total Contribution				50.44%					98.12%	
Total Contribution (only statistically significant values				**52.74%**					**94.53%**	

“In both the logit and probit models all the regressors are involved in computing the changes in probability”

“for the logit model the rate of change in the probability of an event happening is given by ßjPi (1-Pi), where ßj is the (partial regression) coefficient of the jth regressor. But in evaluating Pi, all the variables included in the analysis are involved”

“In the probit model, the rate of change in the probability is somewhat complicated and is given by ßj f (Zi), where f (Zi) is the density function of the standard normal variable and Zi = ß1 + ß2X2i + ... + ßkXki, that is, the regression model used in the analysis”

*SBA* skilled birth assistance, *PNC* postnatal care

In case of SBA, variables that had a statistically significant impact on inequality to access were as follows: wealth inequality (contributed to 23.4% of total inequality in access), inequality in delivery in a private hospital (16.4%), and regional driven inequalities (11%). The inequality in access to the cash-transfer program *Bolsa Familia* appears to have a negative contribution of − 4.4% to overall inequality, favouring the poor. The number of children (3.3%) and living in an urban area (2.9%) had lower contributions to overall inequality in access to SBA. The sum of the contributions of the included variables amounted to 50.44%, resulting in a considerable large error term; this suggests that omitted variables explain a significant portion of inequalities in access to SBA.

In the case of PNC (within 2 months of birth), statistically significant variables included the following: wealth inequality (37.2%), regional driven inequalities (14.7%), inequality in coverage of private health insurance (14%), inequality in mother’s years of schooling (10.4%), and inequality in mother’s age (8.5%). Inequality in delivery by a doctor (4.8%) and living in an urban area (4.5%) contributed to overall inequality, albeit to a lesser extent. The sum of the contributions by the included variables amounted to 98.1%.

It is evident that the chosen variables were better suited for decomposing the socioeconomic-related inequalities of PNC than those of SBA, as it appears when comparing the different elasticities of the variables with regard to SBA or PNC. However, it is necessary to consider that the chosen non-linear model (the probit model in this case) gives only an approximation to the decomposition of inequality, so that the residual component of the model reflects an ‘estimation error’ and an ‘approximation error’ [32].

## Discussion

Several findings stem from our analysis. First, for SBA and ANC, Brazil and Colombia achieved coverage levels above 90% by 2006 and 2010, respectively. Second, our study found high levels of C-section interventions in both countries. Third, there are persistent pro-rich inequities in access to the key interventions, both in Brazil and Colombia. In the case of SBA, ANC, and PNC, normalized CIs suggest that, despite high levels of mean coverage, there seems to be persistent pro-rich inequity of access for these interventions in both countries. Finally, the results of our decomposition analysis of the inequities in access to SBA and PNC in Brazil suggest that wealth inequalities, regional inequalities, inequalities in access to private hospitals, and inequalities in access to private health insurance explain the bulk of the inequities in access.

Some of our results are in line with previous findings, as evidenced by findings regarding access to C-sections. Specifically, mean coverage rates of C-section both in Brazil and Colombia are significantly above the upper limit of 15% recommended by the WHO, suggesting that both countries face difficulties in avoiding unnecessary procedures (such as C-section), which sometimes might lead to worse maternal and newborn health outcomes [31, 33–36]. Several studies have provided evidence for the reasons behind this high rate of C-section, particularly in Brazil. Some of the reasons include the existence of private health insurance, especially for the wealthier people; lack of information regarding the necessity of the intervention; and cultural reasons [36–39]. Our analysis suggests that twice as many women with private health insurance in Brazil opt for C-section, compared to those without private health insurance. The evidence in the Colombian context suggests the significant increase of the C-section rate in Colombia may not only be driven by medical reasons but also by social, cultural, and economic factors [40].

However, our study found that there continue to be pro-rich inequities in access to the key maternal health interventions analysed in this study, both in Brazil and Colombia. This finding has already been documented by several empirical studies [15, 41–43]. More importantly, when observed from the point of view of continuum care for maternal health interventions, the significant pro-rich inequity in PNC is of particular concern, especially given the importance of this intervention for maternal morbidity and mortality outcomes [12].

The decomposition analysis of our study sheds light on the key reasons underlying the persistence of inequities in access to SBA and PNC in Brazil, despite systematic government efforts to eliminate horizontal inequities in access to maternal health services. The decomposition analysis results suggest that wealth inequalities, regional inequality, inequalities in access to private hospitals, and inequalities in access to private health insurance explain the bulk of the inequities in access to both SBA and PNC. Some of the findings, particularly the importance of the regional inequalities, has previously been documented [13, 15, 44]. In addition, the existing evidence suggests that negative perceptions of quality of care have induced women, even those from low-income households, to reject care provided by public health services and to resort to out-of-pocket expenditures to access better quality antenatal care [45]. A significant body of evidence documents issues regarding quality of care; specifically, the rapid increase in health intervention coverage was not coupled with an equally fast improvement in quality [14]. Finally, certain barriers to care related to ethnicity and educational attainment have also been documented [46].

Furthermore, we show that, despite the existence of universal healthcare coverage, Brazil struggles similarly to Colombia—vis-à-vis equitable access to health interventions relevant for maternal care. Formal universal entitlement to healthcare does not always translate into equitable utilization of resources. This is further exacerbated by access to private health insurance. As private health insurance is connected to ability-to-pay and employment status, few individuals are covered by private insurance and thus it tends to be unequally distributed. Hence, the advantage that it provides to those who can afford it tends to exacerbate inequities in access to health services relative to those who must rely on public health services [45].

## Conclusion

The present study constitutes the first attempt to provide a comprehensive analysis of socioeconomic-related inequities in access to maternal health interventions in Brazil and Colombia, by means of a concentration index analysis; furthermore, in the case of Brazil, we employed a decomposition of the concentration index to further study some of the drivers of inequity in access to these interventions. In that respect, the current study sheds light on the causes contributing to the stagnation of progress in reducing maternal mortality.

This study found that significant progress has been made both in Brazil and Colombia in expanding overall access to maternal health interventions, especially for the poorest segments of the population. However, it also found a persistence in pro-rich inequities in access to all four key maternal health interventions in both countries. Contrary to expectations, overall access was less inequitable in Colombia than in Brazil. This result seems counterintuitive, considering that universality of access was not ensured for all Colombian citizens until 2011, whereas in Brazil, universal coverage to comprehensive care is one of the pillars of the system. This finding suggests that formal universal entitlement to health care does not immediately translate into equitable utilization of resources. Further, other barriers to access (e.g. limited offer of services) gain significance when coverage levels are (overall) very high. The decomposition analysis conducted in the Brazilian case provides further evidence to additional barriers to access (e.g. quality of care, availability of private healthcare insurance), which in turn exacerbate the pro-rich inequities—vis-à-vis the key maternal health interventions. Similar findings have been documented, as the barriers of access in Colombia that became prominent as issues of insurance and financial barriers seem to have been, at least, partially resolved.

This study has limitations. The analysis was conducted with cross-sectional data that do not allow an evaluation of the effect of time. Comparisons were made between Brazil and Colombia with data that corresponded to different periods of time. In addition, recall bias has been reported as a significant limitation in similar studies. Finally, certain limitations are due to the use of wealth index, rather than the usual consumption/income measures of socioeconomic status. As noted in Additional file 2, while we tried to make the wealth index as comparable as possible between the two countries, there are slight differences in the questions included in the two surveys (e.g., the Brazilian national DHS does not include questions on cooking fuels). However, given the lack of consumption and income data in the DHS, construction of the wealth index is the best way to gauge socioeconomic status.

Limitations notwithstanding, the findings of this study have important policy implications. Further progress in the reduction of maternal health inequities will require policy initiatives that improve access to high quality services, particularly for the poorer segments of the population. Moreover, addressing inequities in regional and local health infrastructure is crucial. In addition, higher public funding and better management of the public health system could reduce inequalities attributed to the better access of the higher socioeconomic groups to private sector facilities. Finally, the authorities in both Brazil and Colombia should work on curbing the high levels of C-section operations.

## Additional files

Additional file 1: **Table S1.** Summary of Health System Reforms, Policies and Programmes relevant to Maternal Health Services and Outcomes. (DOCX 23 kb)

Additional file 2: Brazil construction of the construction of the wealth index. (DOCX 15 kb)

Additional file 3: Key data summary. (DOCX 13 kb)

## Abbreviations

ANC: Antenatal care
CI: Concentration index
DHS: Demographic and Health Surveys
FP: Family planning
MDG: Millennium Development Goals
MMR: Maternal mortality ratio
PNC: Postnatal care
SBA: Skilled birth assistance
SDG: Sustainable Development Goals
WHO: World Health Organization
