# Supplementary material for: Equity of access to maternal health interventions in Brazil and Colombia: a retrospective study
Source: Int J Equity Health. 2018 Apr 11;17:43. doi: 10.1186/s12939-018-0752-x (PMC5896161; doi:10.1186/s12939-018-0752-x)
Supplement: Supplementary file 1 — Table S1. Summary of Health System Reforms, Policies and Programmes relevant to Maternal Health Services and Outcomes. (DOCX 23 kb) [file 12939_2018_752_MOESM1_ESM.docx]

Appendix 1. Summary of Health System Reforms, Policies and Programmes relevant to Maternal Health Services and Outcomes.
