# Supplementary material for: Equity of access to maternal health interventions in Brazil and Colombia: a retrospective study
Source: Int J Equity Health. 2018 Apr 11;17:43. doi: 10.1186/s12939-018-0752-x (PMC5896161; doi:10.1186/s12939-018-0752-x)
Supplement: Supplementary file 2 — Brazil construction of the construction of the wealth index. (DOCX 15 kb) [file 12939_2018_752_MOESM2_ESM.docx]

***Appendix 2 – Brazil construction of the construction of the wealth index***

To arrive at results that are comparable across the two countries, in the case of Brazil, we constructed a similar asset index as the one that is offered in the case of Colombia and prepared by the DHS team. As in the case in Colombia as well as in Brazil, the asset index was constructed based on information for the following: durable wealth ownership, access to utilities and infrastructure (e.g. sanitation facility and source of water), and housing characteristics (e.g. number of rooms for sleeping and building material), which we included in our analysis.

In constructing the index, we relied on the following questions: (i) the mode of obtaining drinking water to the household; (ii) type of toilet present in the household; (iii) the material used for the walls of the house; (iv) materials used for the floor of the house; (v) ownership of the following items: telephone, electricity, computer, radio, television, bathroom, cars, refrigerators, freezers, aspirators, washing machine, video/DVD. On the basis of the asset index that was created, women were then divided into five wealth quintiles.

It is important to note that while ensuring the above-mentioned wealth index, we obtained results as comparable as possible between the two countries, although there were slight differences in the questions included in the two surveys (for example, the Brazilian national DHS does not include questions on cooking fuels and house ownership). Nevertheless, we believe that we covered the majority of typically used assets when constructing the asset index.
