# Supplementary material for: Equity of access to maternal health interventions in Brazil and Colombia: a retrospective study
Source: Int J Equity Health. 2018 Apr 11;17:43. doi: 10.1186/s12939-018-0752-x (PMC5896161; doi:10.1186/s12939-018-0752-x)
Supplement: Supplementary file 3 — Key data summary. (DOCX 13 kb) [file 12939_2018_752_MOESM3_ESM.docx]

***Appendix 3 – key data summary***

| **Brazilian and Colombian Demographic and Health Survey (DHS) key data** | | | | |  |
| --- | --- | --- | --- | --- | --- |
| **Country** | **Year** | **No. of households** | **No. of eligible women actually interviewed** | **Overall response rate** | **General comments on the survey** |
| **Brazil^1^** | **2006** | 13.613 | 15.575 | 89,2% | Nationally representative household survey with complex probabilistic sampling |
|  |  |  |  |  |  |
|  |  |  |  |  |  |
|  |  |  |  |  |  |
| **Colombia^2^** | **2010** | 51.447 | 53.521 | 93,5% | Nationally representative household survey with complex probabilistic sampling |
|  |  |  |  |  |  |
|  |  |  |  |  |  |
|  |  |  |  |  |  |
| Sources: |  |  |  |  |  |
| 1. Data from Pesquisa Nacional de Demografia e Saúde da Criança e da Mulher. PNDS 2006. Ministério da Saúde. Centro Brasileiro de Análise e Planejamento. Brasília - DF, 2009. | | | | | |
| 2. Data from Apéndice A, B, C Diseño y precisión de la muestra obtained from http://www.profamilia.org.co/encuestas/Profamilia/Profamilia/index.php?option=com_content&view=article&id=152&Itemid=12(accessed on 22th August 2015)  DHS, Demographic Health Surveys | | | | | |
